# Gene expression and histological assessment of capsular fibrosis in post-traumatic shoulder stiffness following plate fixation of proximal humeral fractures: an exploratory pilot study

**DOI:** 10.1186/s12891-026-09944-1

**Published:** 2026-05-08

**Authors:** Leopold Henssler, Daniela Drenkard, Moritz Riedl, Lisa Klute, Felix Keil, Volker Alt, Martijn Riool, Maximilian Kerschbaum

**Affiliations:** 1 Department of Trauma Surgery, University Hospital Regensburg, Franz-Josef-Strauss-Allee 11, 93053 Regensburg, Germany; 2 Department of Pathology, University Hospital Regensburg, Regensburg, Germany

**Keywords:** Proximal humerus, Fracture, Plate fixation, Trauma, Fibrosis, Joint stiffness, Capsular scarring

## Abstract

**Background:**

The molecular and histologic characteristics associated with the development of post-traumatic capsular fibrosis of the shoulder joint following open reduction and internal fixation (ORIF) of proximal humerus fractures (PHF) remain incompletely understood. To help elucidate these mechanisms, this hypothesis-generating pilot study aimed to examine gene expression and histological changes in the joint capsule of patients undergoing implant removal and adhesiolysis for post-traumatic stiffness after surgical fixation of PHF.

**Methods:**

For this pilot study, tissue samples were collected from the joint capsule of patients with a clinical diagnosis of post-traumatic shoulder stiffness during implant removal and adhesiolysis surgery (*n* = 6; median 349 days post-fracture). For comparison, equivalent samples were obtained from patients undergoing fixation of acute proximal humeral fractures (*n* = 5; median 9 days post-fracture). Gene expression analysis of 24 selected genes was performed using RNA extraction, reverse transcription, and quantitative PCR. In addition, histological evaluation was conducted using H&E staining and type V collagen immunohistochemistry.

**Results:**

Exploratory RNA analysis revealed significant upregulation of COL14A1, THBS4, EPHA4, and SIX1 in the post-traumatic stiffness group. Histological assessments demonstrated features of capsular fibrosis, including increased extracellular matrix deposition and disorganized collagen architecture.

**Conclusions:**

RNA analysis on capsular tissue harvested during implant removal in patients with PTSS could be reliably performed. Histology found mature fibrosis while molecular analysis could identify candidate genes as potential target for further research.

**Trial Registration:**

Trial was retrospectively registered at the German Clinical Trials Register (DRKS) on Feb 2nd, 2026 (Registration-ID DRKS00039180.)

**Supplementary Information:**

The online version contains supplementary material available at 10.1186/s12891-026-09944-1.

## Introduction

Proximal humerus fractures (PHFs) rank among the most common fracture sites in adult population [1], especially in the elderly [1, 2]. While in young patients high-energy injuries are the primary cause of PHF, low-energy trauma such as falls from standing height is the most prevalent mechanism in the elderly [3], leading to complex fracture patterns due to reduced bone quality [4]. As demographic shifts contribute to an aging society, the relevance of proximal humerus fractures is anticipated to further increase.

Currently, due to satisfactory functional outcomes and low complication rates reported in large randomized controlled trials [5] and meta-analyses [6], most PHFs are treated nonoperatively, especially in the elderly [7–9]. Nevertheless, surgical treatment with open reduction and locking plate fixation (open reduction and internal fixation; ORIF) remains the preferred treatment modality for more complex displaced fracture types or fracture-dislocations [10, 11], particularly in younger individuals with high functional demands requiring early recovery [12, 13]. Despite advancements in surgical techniques and rehabilitation protocols, the rate of complications and secondary revisions remains high after ORIF [6, 14].

Post-traumatic shoulder stiffness (PTSS) is a common complication after ORIF, occurring in about 24% of cases following PHF [14]. Older studies report more than 50% loss of shoulder function in patients with PTSS despite uneventful bone healing [15], suggesting this condition may contribute to the limited results of ORIF in comparative studies. In contrast to the – etiologically distinct – idiopathic primary adhesive capsulitis (frozen shoulder), secondary post-traumatic shoulder stiffness involves not only contracture of the glenohumeral joint capsule but also periarticular adhesions of the subdeltoid peri-implant tissue and adjacent rotator cuff tendons. Implant removal and open arthrolysis for the treatment of PTSS is frequently performed to improve range of motion [16, 17] and account for more than 50% of revisions after ORIF of PHF [14]. However, open adhesiolysis alone may not adequately address the capsular pathology, which is why additional arthroscopic capsular release after open adhesiolysis and implant removal has shown significant improvements in shoulder function in multiple studies [18–21].

Even though some risk factors for the development of secondary shoulder stiffness following ORIF of PHF have been identified [22, 23], the complex multifactorial interplay of different factors in the pathogenesis of post-traumatic shoulder stiffness after PHF is not fully understood. While existing clinical research focuses on prevention [24] and techniques of surgical removal, particularly of extra-articular adhesions [16, 17], most evidence regarding contracture and fibrosis of the joint capsule itself is derived from in vitro studies and animal experiments. These suggest that an early hyperinflammatory response may be a critical driver of pathological changes within the joint capsule [25]. However, the signalling pathways leading from pro-inflammatory dysregulation to the development of manifest fibrosis have not yet been sufficiently investigated. Understanding these mechanisms may facilitate the development of targeted therapeutic strategies to prevent or treat this condition.

This exploratory, hypothesis-generating pilot study aimed to provide an orientation for characterizing the molecular and histopathological features of capsular tissue in patients with mature secondary PTSS following plate osteosynthesis of proximal humeral fractures, while assessing the feasibility of tissue harvesting and processing. Histological evaluation and gene expression analysis, including RNA extraction, reverse transcription, and quantitative PCR, were performed on capsule samples obtained in the late remodelling phase and compared with samples from patients undergoing ORIF for acute proximal humeral fractures.

## Materials and methods

### Study setting and patient population

This prospective pilot study was conducted at a level I trauma centre in accordance with STROBE guidelines and the Declaration of Helsinki, following approval by the Local Ethics Committee of the University of Regensburg (Ethic Approval Number: 23-3343-101). The study compared two patient groups. Group 1 (post-traumatic shoulder stiffness group; PTSS) included patients diagnosed with mature secondary PTSS undergoing implant removal combined with periarticular adhesiolysis. For availability reasons, the control group in this pilot study consisted of patients undergoing primary surgical fixation for acute PHFs without clinical signs of stiffness (proximal humerus fracture group; PHF). All patients provided written informed consent.

Inclusion criteria required patients in both groups to be at least 18 years old and to provide written informed consent. Patients were excluded if they were under 18 years, lacked consent, or had a history of frozen shoulder or post-traumatic stiffness in any joint. Additional inclusion criteria for the PTSS group included:

- Indication for implant removal and open arthrolysis after plate fixation of the proximal humerus.
- Clinical diagnosis of restricted passive and active range of motion (> 30% loss compared to contralateral side in active abduction, external rotation, and forward flexion).
- Fracture union and no evidence of infection, refracture, or implant failure on plain radiographs of the affected shoulder.

Exclusion criteria for the PTSS group included prior shoulder surgery (other than the index procedure), systemic inflammatory disease, or a history of adhesive capsulitis unrelated to trauma.

### Surgical procedure and tissue sampling

All surgical procedures were performed under general anaesthesia in the beach chair position using a deltopectoral approach. Intravenous antibiotics were administered within 30 min prior to skin incision. In the PHF group, fractures were anatomically reduced and fixed using a titanium alloy locking plate (PHILOS^®^, DePuy Synthes, Johnson & Johnson Medical, Raynham, MA, USA), as previously described [26]. Before wound closure the proximal and caudal border of the subscapularis tendon was clearly identified and, subsequently, a 1 × 1 cm full-thickness tissue sample was collected from the inferior recess of the glenohumeral joint capsule, just beneath the caudal subscapularis tendon border near its insertion at the lesser tuberosity.

In the PTSS group, after skin incision and exposure through the deltopectoral interval, adhesions in the coracoacromial bursa and between the deep surface of the deltoid and the fixation zone were released, followed by excision of the coracoacromial bursa and implant removal. Residual periarticular scar tissue was resected, including debridement of the rotator interval. After adhesiolysis, intraoperative assessment of range of motion was performed. As a last step, the subscapularis tendon was completely exposed, and a comparable full-thickness capsule sample was then obtained using same method as in the PHF control group.

Tissue samples were divided into two parts: one preserved in formalin for histological evaluation, and the other stored in RNAprotect Tissue Reagent (Qiagen, Venlo, Netherlands) immediately after harvesting. All samples were transferred the same day to the Laboratory for Experimental Trauma Surgery for further analysis.

### Clinical evaluation

Passive range of motion for combined glenohumeral and scapulothoracic abduction/elevation was documented according to the neutral-zero method [27]. The range of motion was recorded intraoperatively under anaesthesia, both before and after implant removal and extraarticular arthrolysis.

### RNA isolation, cDNA synthesis, and gene expression analysis

RNA was extracted using a modified protocol with the RNeasy Plus Universal Kit (Qiagen, Venlo, Netherlands). Frozen tissue samples were ground in liquid nitrogen, suspended in TRIzol Reagent (ThermoFisher Scientific, Waltham, MA, USA). The samples were thawed and transferred into an Eppendorf tube. The solution was then divided among Eppendorf tubes (1 mL per tube) and incubated at room temperature for 20 min. Subsequently, 120 µL of gDNA-Eliminator (RNeasy Plus Kit) was added, and the solution was shaken for 30 s. Then, 200 µL of chloroform was added, and the solution was shaken again for 30 s. After incubation for 5 min at room temperature, samples were centrifuged (10,000 × *g*, 12 min, 4 °C). The aqueous phase was carefully collected, an equal volume of TRIzol was added, and after inverting the tube 10 times, the solution was redistributed so that each Eppendorf tube contained a total volume of 1200 µL. The sample was then incubated for 20 min at room temperature. Next, 240 µL of chloroform was added, the solution was shaken for 30 s, incubated for 5 min at room temperature, and centrifuged again (10,000 × *g*, 12 min, 4 °C). The aqueous phase (≥ 600 µL) was then processed using the RNeasy Kit according to manufacturer’s instructions except for the DNase digestion, which was performed directly on the column.

For RNA isolation using the RNeasy Kit, a maximum of 700 µL of the sample was transferred to the RNeasy column per centrifugation step. The column was centrifuged at ≥ 8000 × *g* for 15 s at room temperature, and the process was repeated for the remaining sample. Next, 700 µL of RWT buffer was added, followed by centrifugation at ≥ 8000 × *g* for 15 s, and the flow-through was discarded. DNase digestion was performed directly on the column by preparing a DNase I mixture (10 µL of DNase I stock solution and 70 µL of RDD buffer, Qiagen), mixing gently, and briefly centrifuging if necessary. The mixture was applied to the column and incubated for 15 min at room temperature. The column was then washed by adding 500 µL of RPE buffer, centrifuging at ≥ 8000 × *g* for 15 s, and discarding the flow-through. Another 500 µL of RPE buffer was added, followed by centrifugation at ≥ 8000 × *g* for 15 s, with the flow-through discarded. To remove residual buffer, the column was placed into a new collection tube and centrifuged for an additional 2 min at ≥ 8000 × *g*. Finally, the column was transferred to a new Eppendorf tube, and RNA was eluted using 30–50 µL of DEPC-treated water.

Subsequently RNA was reverse transcribed into cDNA with the Transcriptor First Strand cDNA Synthesis kit (Roche, Basel, Switzerland) according to the manufacturer’s instructions. Quantitative real-time polymerase chain reaction (PCR) was performed using a customized 96-wells plate (PrimePCR™ Primers, Bio-Rad Laboratories, Hercules, CA, USA) and a real-time PCR detection system (CFX96, Biorad Laboratories). A targeted gene expression panel of 24 selected genes (see Supplementary Figure S1) was used to broadly assess biological processes implicated in post-traumatic shoulder stiffness following proximal humerus fracture. The selected genes capture key aspects of tissue remodelling, including extracellular matrix (ECM) composition, fibrotic activation, developmental transcriptional programs, and mechanotransduction, enabling an exploratory, hypothesis-generating analysis of capsular pathology. The primers used can be accessed in Supplementary Table S2. Lastly, gene expression was normalized to the mean expression of each gene in the five samples of the PHF control group. Due to the exploratory pilot nature of this study and in order to identify potential candidate genes associated with capsular fibrosis for further research, the gene expression was analysed without correction for multiple comparisons.

### Qualitative and semi-quantitative histology

Haematoxylin-eosin (H&E) staining and immunohistochemistry were performed for the histological assessment of capsular tissue. After overnight fixation in 4% paraformaldehyde in phosphate buffer, samples were washed in phosphate buffer and cryoprotected in a sucrose gradient (10%, 20%, and 30% sucrose in phosphate buffer). Tissues were then embedded in Tissue-Tek O.C.T. Compound (Sakura Finetec, Torrance, CA, USA) and sectioned at 10 μm thickness using a Leica CM1950 (Wetzlar, Germany) cryostat.

Standard H&E-stained sections were prepared prior to the immunohistochemical analysis. For immunohistochemistry of collagen type V, a rabbit polyclonal anti-collagen type V antibody (ab7046, Abcam, Cambridge, UK) was used. After rehydrating the sections in washing buffer, endogenous peroxidase activity was quenched using 3% H_2_O_2_ and 10% methanol in phosphate-buffered saline (PBS) for 30 min, followed by washing. Antigen retrieval was performed via pepsin digestion for 15 min at room temperature. After three additional washing steps, sections were incubated in blocking buffer (10% goat serum in 0.08 M Tris, 0.8% NaCl) for 60 min at room temperature. This was followed by overnight incubation at 4 °C with the primary antibody diluted in the same blocking buffer.

The next day, sections were incubated for 1 h at room temperature with a biotinylated goat anti-rabbit IgG secondary antibody (Biotin-SP AffiniPure^®^; 111-065-003; Jackson ImmunoResearch, Cambridge, UK) followed by a 90-min incubation with an avidin-biotin complex (VECTASTAIN^®^ Elite^®^ ABC-HRP Kit, PK-6100, Vector Laboratories, Newark, NJ, USA). After 3 washing steps, colour development was performed using diaminobenzidine (DAB; Sigma-Aldrich, St. Louis, MO, USA) and nickel/cobalt enhancement. Stained sections were examined using a Zeiss Axiovert (Oberkochen, Germany) microscope.

To enable a standardized evaluation of joint capsule fibrosis, a semiquantitative, observer-based scoring system was established for the purpose of this study which grades fibrosis on a scale from 0 to 3 based on characteristic histomorphological features, including collagen deposition and organization, cellularity, and reduction of adipose tissue. Histological sections stained with haematoxylin and eosin were evaluated in a blinded manner.

Grade 0 was defined by normal capsular architecture with loosely arranged collagen fibers, physiological cellularity, and preserved adipose tissue. Grade 1 represented mild fibrosis with slightly increased collagen deposition, parallel alignment of collagen fibers, mildly increased cellularity, and minimal reduction of adipose tissue, while overall tissue architecture remained largely intact. Grade 2 was characterized by moderate fibrosis with clearly increased collagen content, variable changes in cellularity, and a noticeable reduction of adipose tissue, accompanied by partial architectural distortion and capsular thickening. Grade 3 indicated severe fibrosis, defined by markedly increased and densely compacted or hyalinized collagen, near-complete loss of adipose tissue, and substantial disruption of normal capsular architecture, with hypocellular fibrotic tissue. Scoring was performed using a pattern-recognition approach, with the final grade reflecting the predominant histological features within each specimen.

### Statistical analysis

Statistical analyses were performed using IBM SPSS Statistics Version 29 (IBM Inc., Armonk, NY, USA), with significance set at *p* < 0.05. Figures were created using GraphPad Prism (GraphPad Software Inc., Boston, MA, USA). Data normality was assessed with the Shapiro-Wilk testing. Since data were not normally distributed, continuous and ordinal variables were presented as medians and ranges. Categorical data were presented as total counts and percentages. The Wilcoxon signed-rank test was used to compare preoperative and postoperative range of motion in the PTSS group. Exploratory group comparisons were performed using the Mann-Whitney U test to generate hypotheses. Analyses of differential expression were adjusted for multiple testing using the Benjamini-Hochberg procedure to control the false discovery rate (FDR) and the adjusted p-values were presented as q-values. Given the limited number of genes analyzed (*n* = 24) and the relatively small sample size, a threshold of FDR < 0.1 was applied. This more permissive cutoff was chosen as a balanced compromise to increase statistical power, reduce the risk of type II errors, and support hypothesis generation for subsequent targeted validation experiments, while still maintaining an expected false discovery proportion of at most 10%.

## Results

### Study population

Six patients were included in the post-traumatic shoulder stiffness (PTSS) group (median age 57 years), and five patients were investigated in the acute proximal humerus fracture (PHF) group (median age 60 years). Patient characteristics were not significantly different between the groups (Table 1). The median time from the initial trauma to intraoperative collection of the investigated tissue samples was heterogenous in the PTSS group but, as expected, significantly longer than in the PHF group (*p* = 0.006). In the PTSS group, implant removal and adhesiolysis resulted in a significant improvement in shoulder abduction (median improvement 35°; range 20° – 55°; *p* = 0.027).

**Table 1:** Patient characteristics in the study groups

	PTSS (*n* = 6)	Acute PHF (*n* = 5)	*p*-value
Sex (m: f)	3:3	3:2	0.752
Age (years)	57 (range 42–63)	60 (range 46–80)	0.582
Time fracture until ORIF (days)	8 (range 1–18)	9 (range 2–18)	0.421
Time fracture until sampling (days)	349 (range 129–575)	9 (range 2–18)	0.006*
Range of Motion			
ABD preoperative	75° (range 30° – 90°)		
ABD postoperative	95° (range 80° – 120°)		0.027*

Data are presented as median values and ranges. *PTSS* post-traumatic stiffness, *PHF * proximal humerus fracture, *ORIF *open reduction and internal fixation, *ABD *abduction

Significant differences in patient characteristics are marked by asterisks (*)

### Gene expression analysis

**Fig. 1:**
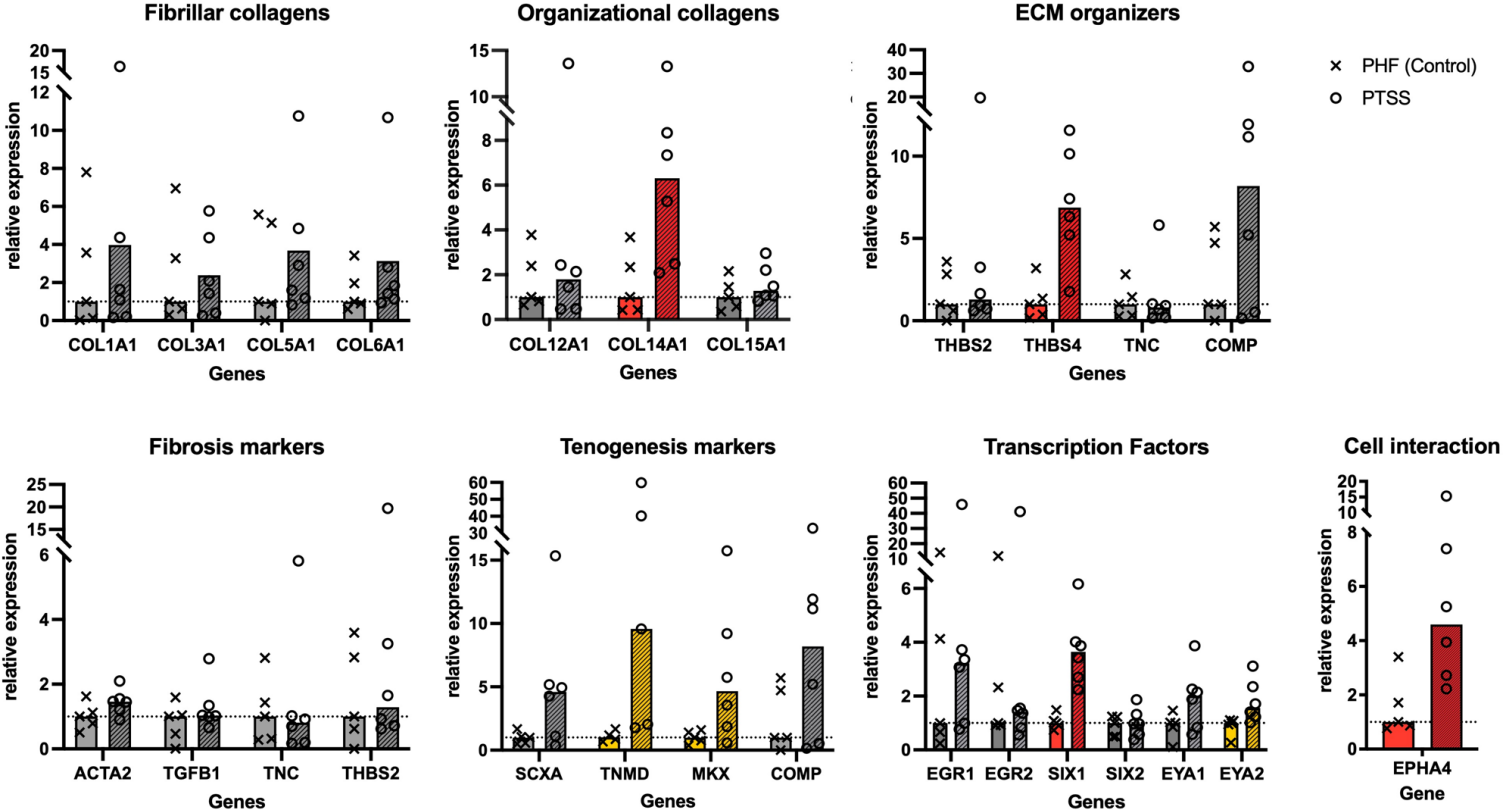
Overview of the expression of the investigated genes. Expression levels are shown as relative expression in the PTSS group (*striped bars*,* circle values*) normalized to the average expression (marked by dashed line) in the acute PHF control group (*clean bars*,* crossed values*). Genes with significantly increased expression before (*yellow*) and after (*red*) correction for multiple comparisons (FDR < 0.1) are highlighted in color. Gene abbreviations are according to the database of the National Center for Biotechnology Information (NCBI)

Seven genes were found to be significantly upregulated before and four genes after correction for multiple comparisons at an FDR < 0.1 (q < 0.1) (Fig. 1; Supplementary Table S3). Structural proteins including type XIV collagen (COL14A1; relative expression 4.86 ± 0.424; *p* = 0.013), Thrombospondin 4 (THBS4; relative expression 7.91 ± 0.40; *p* = 0.005) and Tenomodulin (TNMD; relative expression 9.31 ± 1.05; *p* = 0.033) showed elevated expression. Additionally, regulatory molecules such as Ephrin-receptor A4 (EPHA4; relative expression 3.76 ± 0.42; *p* = 0.010) and transcription factors including Mohawk homeobox protein (MKX; relative expression 4.19 ± 0.70; *p* = 0.036), SIX1 (relative expression 3.50 ± 0.21; *p* < 0.001), and its co-activator EYA2 (relative expression 2.12 ± 0.25; *p* = 0.037) were significantly upregulated. In contrast, α-smooth muscle actin (ACTA2, *p* = 0.095) and collagen types I (COL1A1, *p* = 0.613), III (COL3A1, *p* = 0.947), and V (COL5A1, *p* = 0.315) did not show significant differences between the study groups. After applying the Benjamini-Hochberg correction for multiple comparisons at an FDR threshold of 0.1, only SIX1 (*q* = 0.002), THBS4 (*q* = 0.056), EPHA4 (*q* = 0.077), and COL14A1 (*q* = 0.078) showed significant differences.

### Structural changes in qualitative histological assessment

Capsular soft tissue samples were obtained from similar anatomical regions of the joint following a standardized procedure; all collected specimens consistently represented joint capsule tissue.

**Fig. 2:**
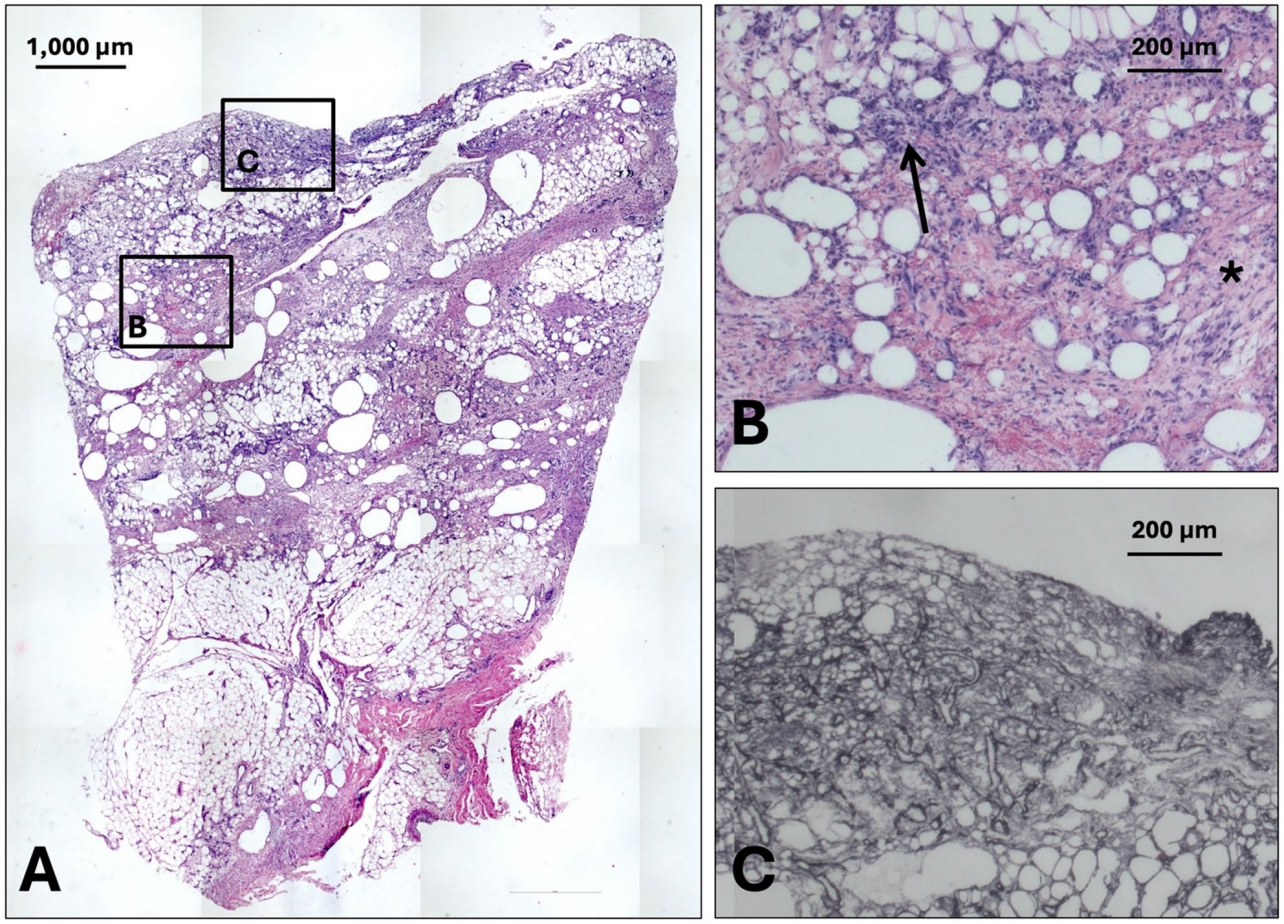
Histology of tissue from a patient with a recent PHF (control group). H&E staining reveals adipose-rich matrix with abundant adipocytes with signs of fat necrosis in the surrounding soft tissue beginning immediately beneath the synovial membrane **(A)** and elevated cellularity **(B)**, especially fibroblast-rich areas (*) and perivascular inflammatory cell infiltrate (**↑**). These regions are characterized by a stroma enriched in collagen V fibers, as demonstrated by immunohistochemistry **(C)**. All specimens are oriented with the synovial side upwards

**Fig. 3:**
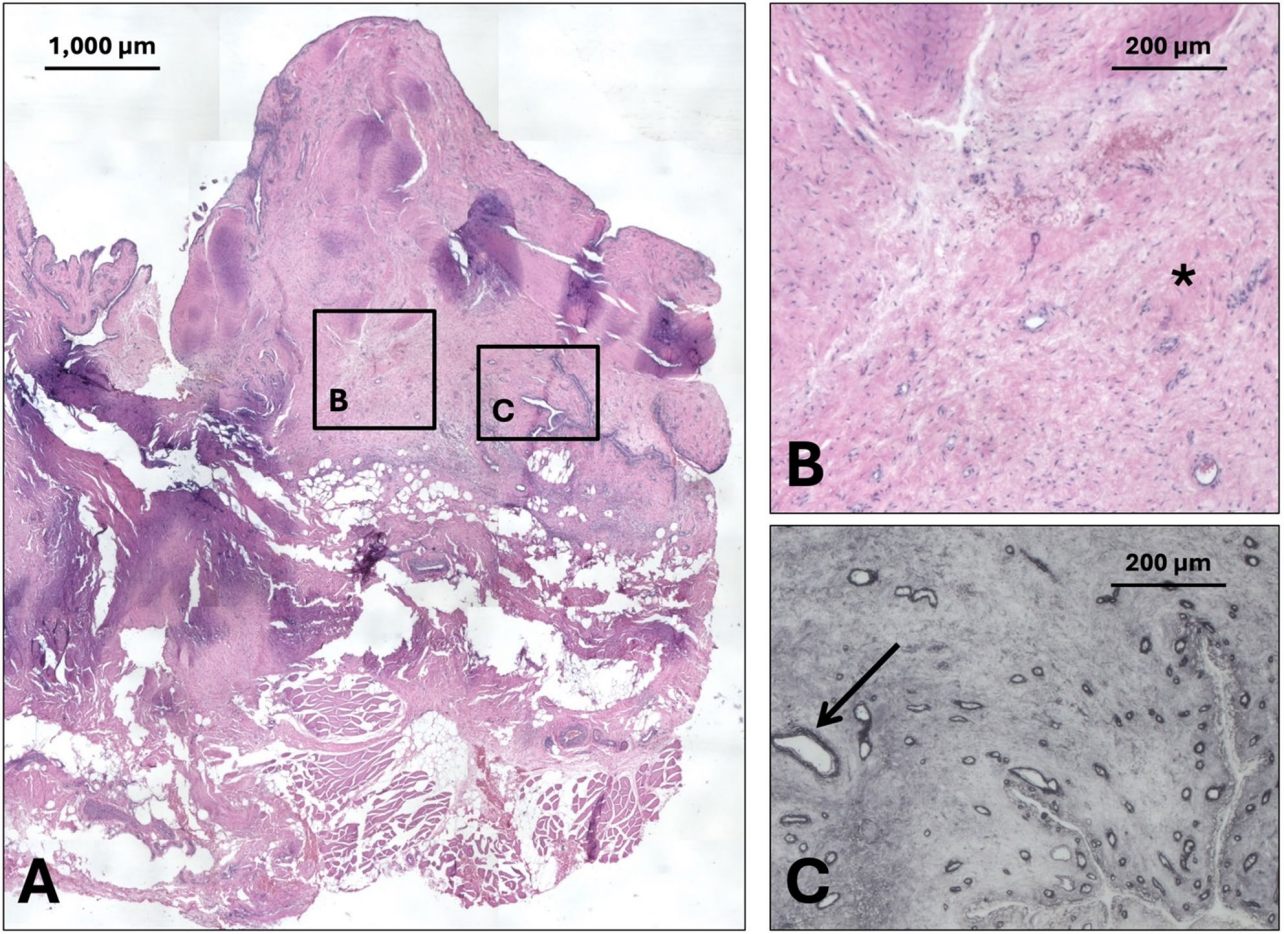
Histology of a post-traumatic stiff shoulder capsule. **A **H&E staining of shoulder capsule tissue from a patient with capsular stiffness demonstrates dense fibrosis with partially hyalinized bundles (*), low cellularity and minimal to absent inflammatory infiltrates, which was characterized as grade 3 according to the semi-quantitative scoring system **(B)**. Immunohistochemistry for collagen V **(C)** shows predominant localization around blood vessels (**↑**), with only sparse collagen V fibers within the fibrotic tissue. All specimens are oriented with the synovial side upwards

Histological analysis revealed marked differences in capsular architecture between the fracture-associated specimen (Fig. 2) and the posttraumatic stiff shoulder capsule (Fig. 3). In the PHF group, the synovial membrane was distinctly thickened and hyperplastic, exceeding the typical 1–3 cell layers, consistent with a reactive synovitis following acute trauma. The underlying subsynovial tissue was characterized by a loose, adipose-rich matrix with abundant adipocytes, interspersed with mildly increased cellularity (Fig. 2B), especially fibroblast-rich areas, and features suggestive of edema and low-grade inflammation. Collagen V immunostaining (Fig. 2C) demonstrated a diffuse distribution within this loosely organized extracellular matrix, supporting the presence of non-fibrotic, remodeling connective tissue. In contrast, the PTSS group exhibited a markedly altered capsular organization. The synovial lining appeared irregular and partially attenuated, without pronounced hyperplasia, indicative of chronic structural remodeling rather than acute inflammation. Most notably, the subsynovial layer was replaced by dense, eosinophilic, collagen-rich fibrous tissue with a near-complete loss of adipose components. The collagen fibers were irregularly oriented in partially hyalinized bundles with reduced cellularity, predominantly composed of spindle-shaped fibrocytes, consistent with a mature scar phenotype (Fig. 3B). Collagen V staining in this specimen was more predominant around blood vessels rather than in the interstitial tissue (Fig. 3C). Using a semi-quantitative observer-based fibrosis grading system the PHF samples were classified as median grade 1 (range 0–1), whereas the samples of the PTSS group were corresponded to grade 3 (range 2–3), based on the extent of collagen deposition, and reduction of adipose tissue (Figs. 2 and 3).

## Discussion

The aim of this exploratory pilot study was to provide initial orientation regarding the molecular and histopathological characteristics of capsular tissue in patients with mature secondary PTSS following plate osteosynthesis of proximal humeral fractures, while assessing the feasibility of tissue harvesting and processing. The following key findings were observed:

- Histology showed pronounced fibrosis, with thickening of the lamina fibrosa beneath the intima, disorganized collagen, and neovascularization.
- RNA analysis of capsule biopsies revealed increased expression of four genes (COL14A1, EPHA4, SIX1, and THBS4) in fibrotic samples.

Tissue healing, for both fractures [28] and soft tissue injuries [29, 30], typically progresses through three phases: an initial inflammatory phase, a proliferative phase, and a remodelling phase. During proliferation, fibroblasts expand, progenitor cells differentiate, and structural collagens and ECM are deposited. In remodelling, the ECM reorganizes into tissue-specific architecture, while cellularity, proliferation, and gene expression decline.

The pathophysiology of PTSS after plating of PHFs is not fully understood. Unlike primary adhesive capsulitis, which is idiopathic and driven by inflammation [31, 32], PTSS appears to result from combined periarticular adhesions and direct capsular scarring [16, 18]. Early postoperative hyperinflammation is thought to trigger later fibrosis. In a rabbit model, Morrey et al. [25] showed that pro-inflammatory cytokines, including IL-1β, IL-6, and IL-8, are upregulated in the joint capsule shortly after injury. The source of this inflammation is unclear. Implant-related immune reactions have been proposed, prompting the development of low cell-adhesive plating materials such as carbon fiber–reinforced polyetheretherketone (CFR-PEEK). However, peri-implant tissue analysis showed higher particle load and inflammation with CFR-PEEK, due to increased wear particle deposition [24].

Based on prior evidence of ventral capsular fibrosis in shoulder stiffness after rotator cuff tears [33], biopsies in this study were taken from the anteroinferior capsule. Samples from the late remodelling phase showed mature fibrosis, with a thickened, disorganized connective tissue layer beneath the intima. In this exploratory pilot study, a targeted gene expression panel was employed to broadly capture key biological processes potentially underlying PTSS. Rather than focusing on a single predefined pathway, the selected genes encompass major axes of tissue remodelling, including extracellular matrix and organization, fibrotic activation and myofibroblast-, tendon- and ligament-associated differentiation, and developmental transcriptional programs. In addition, genes involved in mechanotransduction and cell–cell communication were included to account for altered biomechanical signalling and functional impairment of the joint capsule. This integrative approach was designed to enable hypothesis generation by simultaneously assessing structural, cellular, and signalling pathways, thereby providing a systems-level perspective on maladaptive healing and capsular stiffening.

Interpreting these findings requires considering the temporal dynamics of tissue healing. Most studies on post-traumatic arthrofibrosis, particularly in animal models, focus on the early inflammatory and proliferative phases [25, 34, 35]. In contrast, tissue samples of the present study were obtained at a median of 12 months post-injury, comparable to clinical studies on post-traumatic elbow stiffness [36–38], representing the later healing phase of remodelling. Consistent with this timing, we observed upregulation of genes involved in cell differentiation and intercellular signalling rather than classical markers of proliferation or ECM deposition, which are more characteristic of earlier phases.

Unlike prior reports of sustained collagen types I and III upregulation in chronic post-traumatic elbow stiffness [37], our samples showed no significant increase in these collagens. Instead, collagen type XIV (COL14A1), a fibril-associated collagen typical of mechanically loaded tissues, was significantly elevated. Collagen XIV regulates fibrillogenesis and cross-linking [39] processes associated with joint contractures, and can influence cell proliferation and differentiation [40, 41]. Its overexpression has been described in other fibrotic conditions, although its relative abundance may decline over time [42]. Myofibroblast differentiation, a key driver of arthrofibrosis [36, 38], is marked by alpha-smooth muscle actin (α-SMA, ACTA2). Previous studies [34, 35, 38] show that myofibroblast activity peaks early and declines during remodelling, consistent with our finding of no significant ACTA2 upregulation.

Upregulation of SIX1 indicates activation of developmental transcriptional programs in stiff capsule tissue. SIX1, a homeobox transcription factor, often partners with EYA2 to regulate proliferation, cell fate, and differentiation in muscle and other mesenchymal lineages [43]. EYA2 acts as a co-activator, enhancing SIX1-driven transcription and influencing pathways such as TGF-β [44]. Their aberrant expression may promote fibroproliferation and ECM remodelling, contributing to pathological contracture in late PTSS.

TGF-β is a well-established profibrotic mediator [45, 46], but broad inhibition is clinically impractical due to its pleiotropic effects. Targeting downstream effectors, such as the Ephrin signalling pathway, may offer a more feasible approach. Notably, EPHA4, encoding the Ephrin-A4 receptor, was overexpressed. Ephrin-Eph signalling modulates cytoskeletal remodelling, cell adhesion, and ECM organization in tissue repair [30, 47] and EPHA4 is linked to macrophage-driven inflammation in other fibrotic pathologies [48, 49]. In a murine model, lung fibrosis could be induced by Ephrin injections, whereas pharmacologic Ephrin inhibition significantly attenuated fibrotic changes [50]. These findings highlight Ephrin signalling as a potential target for further research and therapeutic approach also in arthrofibrosis.

Thrombospondin-4 is known to be involved in tissue regeneration and ECM remodelling during wound healing by regulation of collagen fibrillogenesis and increasing collagen fibre density by binding other structural proteins [51]. In accordance with the presented results its upregulation has also been observed in other fibrotic pathologies, such as hypertrophic scars following burn trauma [52].

These findings should be interpreted in light of several limitations. First, gene expression was assessed at a late time point, reflecting the residual transcriptional profile of the remodeling phase rather than the dynamic early post-traumatic response [25]. Protein-level analyses, which may be more informative at this stage, were not performed in this pilot study but are planned for future investigations. We also explicitly acknowledge the absence of negative controls in the immunohistochemical analysis as a methodological limitation.

Second, the control group has inherent limitations, as gene expression is naturally elevated during early fracture healing. Therefore, some observed differences may reflect healing-phase variation rather than fibrosis-specific mechanisms, although they still provide insight into mature capsular fibrosis. A more appropriate control group would include patients undergoing implant removal without post-traumatic stiffness. However, such cases are rare in the authors’ clinical setting. Future studies will aim to include this comparison group.

Additionally, the analysis was limited to a heterogeneous panel of 24 genes and therefore does not capture the full molecular spectrum of post-traumatic fibrosis. The FDR threshold was set at 0.1, which is more permissive than the conventional 0.05. This decision was made because of the small number of genes tested and the limited statistical power of this exploratory pilot study. Applying a stricter threshold would have substantially reduced the number of candidate genes and risked overlooking potentially relevant targets. Given the hypothesis-generating nature of this analysis, an FDR < 0.1 provided a pragmatic balance between sensitivity and control of false-positive findings, facilitating hypothesis generation for future validation studies.

In conclusion, a hypothesis-generating exploratory pilot study was performed, which demonstrated that reliable RNA analysis of capsular tissue obtained during implant removal in PTSS patients is feasible. At a median of twelve months post-injury, molecular and histological findings indicated advanced fibrosis. Consistent with the remodelling phase of the physiologic healing cascade, regulatory genes such as EPHA4 and THBS4 emerged as potential candidates for further study. Future research is necessary and should assess more detailed transcriptomic analyses and protein-level changes across time points with appropriately matched controls to validate these findings and identify targetable pathways for prevention and treatment of PTSS.

## Supplementary Information

Supplementary Material 1.

## Data Availability

The data supporting the findings of this study will be made available to interested researchers upon a reasonable request for a period of five years.

## Abbreviations

ABD: Abduction of the shoulder
CFR-PEEK: Carbon fiber-reinforced polyetheretherketone
DNA: Deoxyribonucleic acid
ECM: Extracellular matrix
FDR: False discovery rate
H&E: Haematoxylin-eosin
NCBI: National Center for Biotechnology Information
ORIF: Open reduction and internal fixation
PHF: Proximal humeral fracture
PTSS: Post-traumatic shoulder stiffness
(q)PCR: (quantitative) Polymerase chain reaction
RNA: Ribonucleic acid
SEM: Standard error of the mean
STROBE: STrengthening the Reporting of OBservational studies in Epidemiology
