# Supplementary material for: Gene expression and histological assessment of capsular fibrosis in post-traumatic shoulder stiffness following plate fixation of proximal humeral fractures: an exploratory pilot study
Source: BMC Musculoskelet Disord. 2026 May 8;27:404. doi: 10.1186/s12891-026-09944-1 (PMC13159271; doi:10.1186/s12891-026-09944-1)
Supplement: Supplementary file 1 — Supplementary Material 1. [file 12891_2026_9944_MOESM1_ESM.docx]

**Supplements:**

**Supplementary Figure S1: Schematic illustration of the PCR plate configuration.**


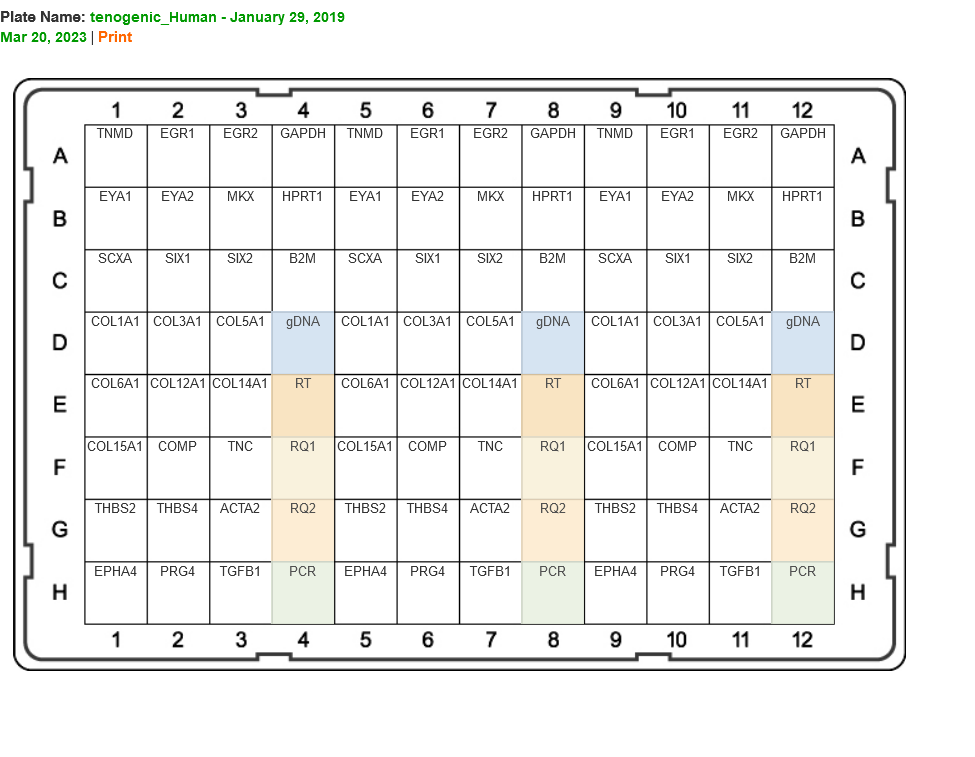


**Supplementary Table S2: Primer List**

| ***GENE*** | ***PROBE ASSAY*** | ***SEQUENCE*** |
| --- | --- | --- |
| **ACTA2** | qHsaCIP0028813 | GCGGCAGTGGCCATCTCATTTTCAAAGTCCAGAGCTACATAACACAGTTTCTCCTTGATGTCCCGGACAATCTCACGCTCAGCAGTAGTAACGAAGGAATAGCCACGC |
| **COL12A1** | qHsaCEP0050667 | GATTCAAAGTCGTCCACAATGAACACGTGCCGTTCACTTGGTTTGCTGGCAATGTTGGCAAGCTCATTGTAGTCGACATCAGCCACACCAACTACAAAGACAC |
| **COL14A1** | qHsaCEP0055198 | GCTTCTGACCTTCTACTGTACGACGTGACTGAGAACAGCATGCGAGTCAAATGGGATGCAGTGCCTGGGGCCTCAGGTTACCTGATCCTTTATGCTCCTCTAACAGAGGG |
| **COL15A1** | qHsaCIP0027564 | CCCAAAGAAGCAAAAGTTGAACCCATAAACACACCTCCAACTCCATCCTCCCCCTTTGAAGACATGGAACTTTCTGGTGAACCTGTACCCGAGGGGACCCTGGAAACCACCAACATGAGCATCATCCAGCACAGCAGCCCCAAACAAGGGTCTGGTGAGATCCTGAATGACACACTGGAG |
| **COL1A1** | qHsaCEP0050510 | TCTTGGTCTCGTCACAGATCACGTCATCGCACAACACCTTGCCGTTGTCGCAGACGCAGATCCGGCAGGGCTCGGGTTTCCACACGTCTCGGTCATGGTACCTGAGGCCGTTCTGTACGCAGGTGATTGGTGGGATGTCTTCG |
| **COL3A1** | qHsaCEP0053821 | ACACCGATGAGATTATGACTTCACTCAAGTCTGTTAATGGACAAATAGAAAGCCTCATTAGTCCTGATGGTTCTCGTAAAAACCCCGCTAGAAACTGCAGAGACCTGAAATTCTGCCATCCTGAACTCAAGAGTGGA |
| **COL5A1** | qHsaCIP0029421 | AAGAGATCTTCGGCTCTCTCAACTCTCTGAAGCTGGAGATTGAGCAGATGAAACGGCCCCTGGGCACGCAGCAGAACCCCGCCCGCACCTGCAAGGACCTGCAGCTCTGCCACCCCGACTTCCCAGATGGTGAATACTGGGTCGATCCTAACCAAGGATGCTCCAGGGATTCCTTCAAGGTTTACTGCAACTTCACAGCCGGGGGGTCGACATGCGTCTTCCC |
| **COL6A1** | qHsaCEP0052310 | ACCTGAAGGAGAATAAGTACCTGATTGTGGTGACCGACGGGCACCCCCTGGAGGGCTACAAGGAACCCTGTGGGGGGCTGGAGGATGCTGTGAACGAGGCCAAGCACCTGGGCGTCAAAGTCTTCTCGGTGGCCATCACACCCGACCA |
| **COMP** | qHsaCIP0032897 | TATACCATCGCCATCACTGTCCTTCTGGTCTGAGTTGGGTACCCTAGGGCAGTTGTCGGCCTGGTTGCGGATCCGGTCGCCGTCGATGTCGTCGTCGCACGCATCGCCCCGGCCGTCCTGGTCTGTGTCCTTTTGGTCGTCGTTCTTCTGGGACCGGCAGTTGTCGCACGCATCGCCC |
| **EGR1** | qHsaCEP0039196 | CCACTCGACTGCCCCCCATCACCTATACTGGCCGCTTTTCCCTGGAGCCTGCACCCAACAGTGGCAACACCTTGTGGCCCGAGCCCCTCTTCAGCTTGGTCAGTGGCCTAGTGAGCATGACCAACCCACCGGCCT |
| **EGR2** | qHsaCIP0039206 | TGCCCATGTAAGTGAAGGTCTGGTTTCTAGGTGCAGAGACGGGAGCAAAGCTGCTGGGATATGGGAGATCCAACGACCTCTTCTCTCCAGTCATGTCAATGTTGATCATGCCATCTCCGGCCACTCCGTTCATCTGGTCAAAGGGGCCTC |
| **EPHA4** | qHsaCIP0031570 | CTGGCGGCCACCTGTATTCTGAGGGCTACTCCATTCCAAGTTCACAGATGTCTCGTTGACATTTGAAATCAAGTTCAGGGGAGCAGATGGTGGACGGGTGCAGGGCATAGAGGCAGCATCGTTGTCAGCTCTGAAAAAGC |
| **EYA1** | qHsaCIP0026452 | GTGATTTCCCATCTGAACCTCGACGCAATCGATCAGAATCTGAATCTTTAATGGGTGTTGATGGGCTGTGGATTGTGCTGTACTCTGCTGTGGGATCTGTAACTGCTTGGCTGGTGATGCCAGATGGCGGTTCTTGAAGCTGGTAAGTGGCATTGGTGGATGGTGTCG |
| **EYA2** | qHsaCIP0027167 | AGCCCATACACCTACCAGATGCACGGCACAACAGGGTTCTATCAAGGAGGAAATGGACTGGGCAACGCAGCCGGTTTCGGGAGTGTGCACCAGGACTATCCTTCCTACCCCGGCTTCCCCCAGAGCCAGTACCCCCAGTATTACGGCTCATCCTACAACCCTCCCTACGTCC |
| **MKX** | qHsaCIP0027196 | GTATTCTTAAGCCGACGTCTTGCATTAGCAAACCAATTTGACACCTGCACTAGCGTCATCTGCGAGCCGAGGGCCAAGAGTATCTTCTCGGTCTTGGTGGGGTACGGGTTGTCACGGTG |
| **PRG4** | qHsaCEP0050955 | TGTAGATGAAGCTGGAAGTGGATTGGACAATGGTGACTTCAAGGTCACAACTCCTGACACGTCTACCACCCAACACAATAAAGTCAGCACATCTC |
| **SCXA** | qHsaCEP0041824 | CGCTGATCCCCACCGAGCCCGCCGACCGCAAGCTCTCCAAGATTGAGACGCTGCGCCTGGCCTCCAGCTACATCTCGCACCTGGGCAACG |
| **SIX1** | qHsaCEP0039230 | ATTTGTGAAAGTCCACCATTCCTTTATGCGCAAACAACTCCAGAAACAAGCTGCAAAAATGTTCCTGATTTCTATTTACAAGTGTCCCTAGTCGCTGCAGTGGTTGCTGCTCCAG |
| **SIX2** | qHsaCEP0053978 | GGACACAGAGTACAAGAGACTGGCAGGAGAGAAGAGAGGAGAAACAGAGAAGGAGAGAGAAAGGGAGAGACAGAAGGAGAGAATGAACGGTGGCAAGC |
| **TGFB1** | qHsaCIP0030973 | AACCACTGCCGCACAACTCCGGTGACATCAAAAGATAACCACTCTGGCGAGTCGCTGGGTGCCAGCAGCCGGTTGCTGAGGTATCGCCAGGAATTGTTGCTGTATTTCTGGTACAGCTCCACGTGCTGCTCCACTTTTAACTTGAGCCTCAGCAGACGCAGCTCTGCCCGGGA |
| **THBS2** | qHsaCIP0028253 | CCCATCAATGTCCACGGAGCAGGCGTCACCCTCTCCATTGTTGTCTGTGTCGATCTGGGCAGGGTTGTGCACGTAAGGGCAGTTGTCACAGCGGTCCCCAACCTCATCCTTGTCATAGTCAGCCTGGCGGGGATTGAAGAGGAGCTGGCAGTTGTCCTTCTCATCGGTCACACCGT |
| **THBS4** | qHsaCEP0050746 | TGACTTCGAAGGGACCTTCCATGTGAATACCCAGACAGATGATGACTATGCAGGCTTTATCTTTGGCTACCAAGATAGCTCCAGCTTCTACGTGGTCATGTGGAAGCAGACGGAGCAGACATATTGGCAAGCCACCCCATTCC |
| **TNC** | qHsaCEP0058481 | CCTGTGTAGGAGATGACATAACTGTCCACAGTGGCAATGGCTGGCTGCCACCTGGCCAAGGCTTCTGAGTCAGTGATGTTGGCTGTCACCAGGCCAGATGGGCCATCCAGAG |
| **TNMD** | qHsaCIP0029219 | GGAGATTTGTGATAACGTGACCATGTATTGGATCAATCCCACTCTAATATCAGTTTCTGAGTTACAAGACTTTGAGGAGGAGGGAGAAGATCTTCACTTTCCTGCCAACGAAAAAAAAGGGATTGAACAAAATGAACAGTGGGTGGTCCCTCAAGTGAAAGTAGAGAAGACCCGTCACGCCAGACAAGCAAGTGAGGAAGAACTTCCAATAAATGACTATACTGAAAATG |

**Supplementary Table S3: Relative expression of the investigated genes.**

| **Gene** | **Protein** | **Relative expression (mean ± SEM)** | **Raw  p-value** | **q-value (FDR adjusted)** | **Significant at FDR < 0.1** |
| --- | --- | --- | --- | --- | --- |
| ACTA2 | α-smooth muscle actin | 1.49 ± 0.17 | 0.0950 | 0.2535 | not significant |
| COL12A1 | Collagen type XII | 1.23 ± 0.73 | 0.7506 | 0.9192 | not significant |
| **COL14A1** | **Collagen type XIV** | **4.86 ± 0.42** | **0.0130** | **0.0780** | **significant** |
| COL15A1 | Collagen type XV | 1.58 ± 0.29 | 0.2391 | 0.4782 | not significant |
| COL1A1 | Collagen type I | 1.86 ± 1.03 | 0.6129 | 0.8172 | not significant |
| COL3A1 | Collagen type III | 1.05 ± 0.74 | 0.9472 | 0.9489 | not significant |
| COL5A1 | Collagen type V | 3.56 ± 0.56 | 0.3149 | 0.5039 | not significant |
| COL6A1 | Collagen type VI | 1.62 ± 0.52 | 0.3503 | 0.5255 | not significant |
| COMP | Cartilage oligomeric matrix protein | 7.58 ± 1.21 | 0.2737 | 0.5039 | not significant |
| EGR1 | Early growth response protein 1 | 2.12 ± 0.85 | 0.4349 | 0.6140 | not significant |
| EGR2 | Early growth response protein 2 | 1.06 ± 0.91 | 0.9489 | 0.9489 | not significant |
| **EPHA4** | **Ephrin A4** | **3.76 ± 0.42** | **0.0096** | **0.0767** | **significant** |
| EYA1 | Eyes absent homolog 1 | 2.39 ± 0.41 | 0.1311 | 0.3145 | not significant |
| EYA2 | Eyes absent homolog 2 | 2.12 ± 0.25 | 0.0372 | 0.1274 | not significant |
| MKX | Mohawk homeobox protein | 4.19 ± 0.70 | 0.0363 | 0.1274 | not significant |
| PRG4 | Proteoglycan 4 (Lubricin) | 0.68 ± 1.50 | 0.8043 | 0.9192 | not significant |
| SCXA | Scleraxis A | 3.44 ± 0.77 | 0.0801 | 0.2403 | not significant |
| **SIX1** | **Homeobox protein SIX1** | **3.50 ± 0.21** | **0.0001** | **0.0025** | **significant** |
| SIX2 | Homeobox protein SIX2 | 1.09 ± 0.34 | 0.7895 | 0.9192 | not significant |
| TGFB1 | Transforming growth factor-β1 | 3.24 ± 0.29 | 0.2265 | 0.4782 | not significant |
| THBS2 | Thrombospondin 2 | 5.23 ± 0.77 | 0.2966 | 0.5039 | not significant |
| **THBS4** | **Thrombospondin 4** | **7.91 ± 0.40** | **0.0047** | **0.0560** | **significant** |
| TNC | Tenascin | 0.88 ± 0.76 | 0.8561 | 0.9339 | not significant |
| TNMD | Tenomodulin | 9.31 ± 1.05 | 0.0333 | 0.1274 | not significant |

Genes significantly upregulated in PTSS cases are shown in bold. Data are presented as mean values and standard error of the mean (SEM). Gene abbreviations are according to the database of the National Center for Biotechnology Information (NCBI).
